# Screening for Precancerous Lesions of Upper Gastrointestinal Tract: From the Endoscopists' Viewpoint

**DOI:** 10.1155/2013/681439

**Published:** 2013-03-19

**Authors:** Chen-Shuan Chung, Hsiu-Po Wang

**Affiliations:** ^1^Department of Internal Medicine, National Taiwan University Hospital, College of Medicine, National Taiwan University, Chung-Shan South Road, Taipei 100, Taiwan; ^2^Department of Internal Medicine, Far Eastern Memorial Hospital, Banciao District, New Taipei City, Taiwan

## Abstract

Upper gastrointestinal tract cancers are one of the most important leading causes of cancer death worldwide. Diagnosis at late stages always brings about poor outcome of these malignancies. The early detection of precancerous or early cancerous lesions of gastrointestinal tract is therefore of utmost importance to improve the overall outcome and maintain a good quality of life of patients. The desire of endoscopists to visualize the invisibles under conventional white-light endoscopy has accelerated the advancements in endoscopy technologies. Nowadays, image-enhanced endoscopy which utilizes optical- or dye-based contrasting techniques has been widely applied in endoscopic screening program of gastrointestinal tract malignancies. These contrasting endoscopic technologies not only improve the visualization of early foci missed by conventional endoscopy, but also gain the insight of histopathology and tumor invasiveness, that is so-called optical biopsy. Here, we will review the application of advanced endoscopy technique in screening program of upper gastrointestinal tract cancers.

## 1. Introduction

Malignancies of gastrointestinal tract represent the leading cause of cancer death worldwide. Esophageal and gastric cancers, which have an overall 5-year survival rate of 10~20% and 20~30%, respectively, comprise the majority of the upper gastrointestinal (UGI) tract malignancy [1, 2]. Barrett's esophagus (BE) was regarded as the premalignant lesion for esophageal adenocarcinoma (EAC) based on the unmasking of underlying intestinal metaplasia mucosa after chemotherapy [3]. Moreover, a retrospective study has shown high prevalence of BE in pretreatment (75%) and postchemotherapy (97%) EAC patients [4]. However, emerging evidence has shown that the risk of EAC in BE patients was not as high as expected previously [5]. Recently, a nationwide population-based cohort study disclosed that the annual risk of EAC was 0.12% among patients with BE (increased to 0.26% when high-grade dysplasia was taken into account) [5]. The results call into question the rationale for periodically endoscopic surveillance in BE patients without dysplasia. Unlike the higher proportion of the histology subtype adenocarcinoma of the esophagus in Western countries, approximately over 90% of the esophageal cancer in countries located at the “esophageal cancer belt,” which stretches from Caspian Sea across Central Asia to the West Pacific, are esophageal squamous cell carcinomas (ESCC) [1]. The global incidence of esophageal cancer increased gradually probably because of increasing prevalence of the BE and the habits of psychoactive substance use, such as cigarettes smoking, alcohol consumption, and betel quid chewing, which cause ESCC [1]. Conversely, a steady declining incidence of gastric cancer in many countries has been observed in the last few decades probably because of improved sanitation and *Helicobacter pylori* (Hp) eradication therapy [1, 2]. Hp infection, atrophic gastritis, and intestinal metaplasia are among the most important premalignant conditions of gastric cancer [1, 6]. In patients at risk, annual surveillance can detect tumors at an earlier stage (stage I and II 67% versus 23%; *P* < 0.05) with a major improvement in 5-year survival (50% versus 10%; *P* = 0.006) when compared with open access study population [6]. The UGI tract cancer remains imposing considerable impacts on the public health.

The outcome of UGI tract cancer is closely associated with the stage at diagnosis. The poor prognosis of UGI tract cancer is largely attributed to the delay diagnosis at symptomatic conditions. Nevertheless, the 5-year survival rate for superficial ESCC and early gastric cancer (EGC) may exceed 80% and 90%, respectively [1, 7–9]. Based on an investigation of 290 surgically resected ESCC cases, cancers invading epithelium and lamina propria did not have lymph node (LN) metastasis, and a 5-year survival rate for surgical resected cancers limited to mucosa, and superficial submucosa was 100% [9]. The accumulated experience has also shown that EGC has lower risk for LN metastasis. The analysis of over thousands of gastric cancer patients who had undergone gastrectomy and LN dissection has suggested the low risk of LN metastasis for well-differentiated intramucosal adenocarcinoma without ulcer findings irrespective of the tumor size and those less than 3 cm with ulcer findings [10, 11]. Neither of well-differentiated EGC invading superficial (<500 *μ*m) submucosa with tumor size less than 3 cm and undifferentiated intramucosal EGC less than 2 cm without ulcers have LN metastasis risk [10, 11]. The clinical experiences and data disclosed excellent survivals for these early UGI tract cancers as long as the potential for LN metastasis can be excluded [1, 2, 7, 9].

In the last few decades, endoscopists try to use minimally invasive procedures to remove the superficial UGI tract cancers without concern of LN metastasis. The survival of early cancers after endoscopic removal with organ preservation could be similar to those after surgical resection. Endoscopic removal of EGC was first described in 1974, and endoscopic mucosal resection (EMR) technique with “strip-biopsy” method was published in 1984 [12, 13]. In the following years, different EMR techniques with improvement in equipment accessories, such as cap and ligation method, have been introduced [14, 15]. However, the *en bloc *resection rate using EMR methods was low, especially for those larger than 2 cm or with invasion deeper than submucosa layer which were prone to recur locally even after piecemeal EMR [13, 16]. To achieve minimal invasive *en bloc* resection and provide intact specimen for pathological examination, endoscopic submucosal dissection (ESD) becomes a safe alternative for endoscopic management of early UGI tract cancers [1, 13, 16, 17]. The *en bloc *rate and recurrence rate of ESD for early ESCC were about 95% and 0%, respectively [16]. Similarly, the *en bloc *rate for EGC in the ESD group was significantly higher than that in EMR group (odds ratio (OR) 9.69; 95% confidence interval (CI), 7.74–12.13), and the ESD group had lower recurrence rate (OR 0.10; 95% CI, 0.06–0.18) [18]. Therefore, since the early 2000s, ESD has been regarded as a treatment alternative for early UGI tract cancers in most Asian countries, including Japan, Korean, China, and Taiwan.

Given the high chance for curative treatment of early UGI tract cancers by endoscopic resection with intents of minimal invasiveness, a detailed and thorough endoscopic examination for precancerous or early cancerous lesions is of paramount importance to improve the overall outcome. Here, we will review the literatures on the surveillance of precancerous lesions of UGI tract from the endoscopist viewpoint.

## 2. Image-Enhanced Endoscopy Screening of Upper Gastrointestinal Tract Cancers

The subtle change from precancerous or early cancerous UGI tract cancers is always challenging to endoscopists when using conventional white-light imaging (WLI) endoscopy. To enhance the contrast and resolution of endoscopic images, advances in biomedical optics and endoscopic equipments have been made rapidly in the latest decade. The image-enhanced endoscopy (IEE) system utilizes different enhancing methods by means of dye, optical, and electronic contrasting to allow improved visualization of precancerous lesions and to gain insight of the pathology and invasiveness of the lesions [19]. The techniques and applications of IEE in surveillance of UGI tract cancer will be described.

### 2.1. The Principles of IEE for UGI Tract

#### 2.1.1. Narrow-Band Imaging System with Magnifying Endoscopy

**Figure 1:**
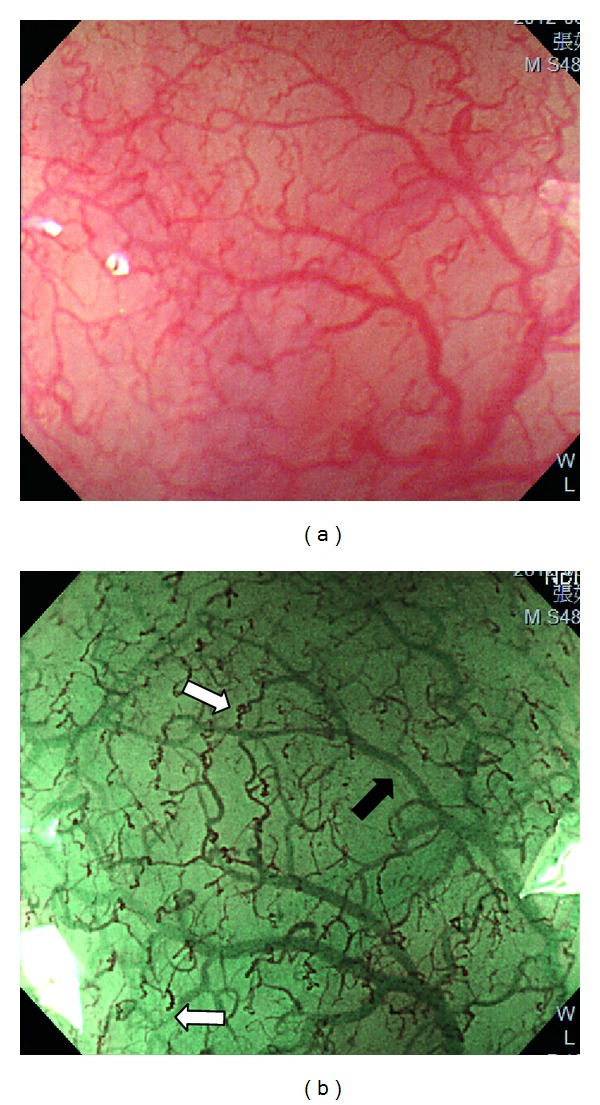
(a) Magnifying endoscopy under white-light imaging shows microvasculature of the normal esophagus. (b) Magnifying endoscopy under narrow-band imaging improves visualization of the reddish-brown superficial vessels (white arrow, Inoue's classification of intraepithelial papillary capillary loops type II) and the cyanic hue deeper vessels (black arrow).

**Figure 2:**
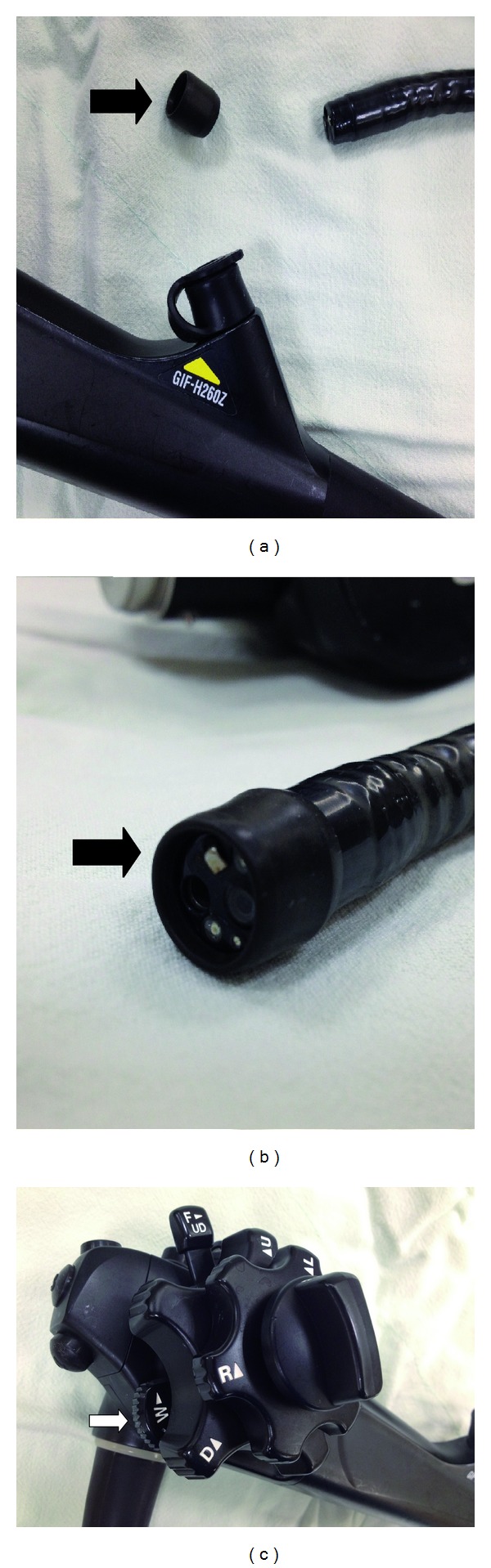
By adjusting a switchover apparatus (white arrow), the magnifying endoscope (GIF-H260Z, Olympus Medical Systems Corp, Tokyo, Japan) with plastic cap-fitted at its end (black arrow) can provide 80-fold zooming images.

 The development of spectroscopy began over 10 years ago. Many efforts have been made to find the best specific pattern of spectrum for excellent enhancement of mucosa surface and microvascular architecture. The narrow-band imaging (NBI) system which was developed since 1999 and commercialized available in 2005. In NBI system, an optic filter is used to illuminate lights with 400–430 nm and 525–555 nm narrowed wavelengths instead of red-green-blue (RGB) broadband light which is utilized in conventional WLI system. Based on the characteristics of light absorption and scattering, the NBI system can accentuate the mucosa surface and vessels at different depths [20]. Angiogenesis is one of the subtle histological changes during carcinogenesis. The hemoglobin which can be considered as a chromophore associated with angiogenesis plays an important role in image formation of living tissues. The GB light is well absorbed by the hemoglobin, thereby contrasting well the morphology of vessels at different depths. The superficial vessels absorb light, with shorter wavelength and become reddish brown, which is the complementary color of GB light, whereas the deeper vessels absorb light with longer wavelength and appear cyanic hue (Figure 1). It is most appropriate to use 415 ± 15 nm blue light and 540 ± 15 nm green light to observe mucosal surface microvasculature and deeper submucosal vessel, respectively. Magnifying or zoom endoscope has been developed over 40 years [21]. Combined with NBI system, the magnifying endoscope (ME) which has the ability to magnify the image to 150x with minimal discriminating diameter about 10 *μ*m can detect precancerous or early cancerous lesions more accurately [22, 23]. For the well-fixed magnified images, a black rubber or transparent plastic hood is crucial to maintain a consistent distance of 2-3 mm between tissues and endoscopic camera lens (Figure 2). The microvascular architecture and the invasiveness of neoplasm could be well delineated by the ME-NBI [22, 24, 25]. 

#### 2.1.2. Flexible Spectral Imaging Color Enhancement

Flexible spectral imaging color enhancement (FICE), or optical band imaging, is a dyeless optical contrast technique based on spectral estimation technology. Ordinary endoscopic pictures are taken by charge-coupled device camera in a regular endoscopy and arithematically processed. Different from NBI system with fixed wavelengths, composite FICE images are generated on a computer which allows viewing of an image taken under light at any suitable wavelength setting for specific condition [26, 27]. Between wavelength 400 nm and 695 nm of visible light, 60 spectral images at 5 nm interval can be selected. The digital processing system can make switchover between ordinary image and FICE image faster than the NBI system which uses the optical filter. Using unlimited combinations of selected spectral transmittance with dedicated wavelengths, the FICE system is useful in discriminating among nonneoplastic and neoplastic lesions of the UGI tract [28].

#### 2.1.3. Autofluorescence Imaging System

 Autofluorescence imaging (AFI) system produces real-time images by the detection of changes in autofluorescence of malignant tissues. The AFI system can detect the differences of concentration or depth distribution of endogenous fluorophores, such as collagen, nicotinamide, adenine dinucleotide, flavin, and porphyrins, between normal and cancerous tissues [29, 30]. The autofluorescence with longer wavelength is emitted by excitation short-wavelength blue light (395–475 nm). The intensity of autofluorescence, green (550 nm) and red (610 nm) reflectance images, was provided for pseudocoloring. An image processor makes autofluorescence images to green color, the green reflectance image to red color, and the red reflectance image to blue color, then AFI pseudocolored images were composited. The difference in autofluorescence emission between normal and dysplastic/cancerous mucosa is likely due to changes in nuclear/cytoplasmic (N/C) ratio and concentrations of collagen and hemoglobin. Normal mucosa emits brighter autofluorescence than cancerous parts, thus, the composite color appears greenish. Because hemoglobin absorbs both autofluorescence and green light (550 nm), vessels or inflammatory mucosa that contain more hemoglobin were displayed as bluish. Because autofluorescence is absorbed well by dysplastic/cancerous mucosa, tumor parts appear magenta in the AFI image [29].

#### 2.1.4. Confocal Laser Endomicroscopy and Endocytoscopy

 Confocal laser endomicroscopy (CLE) was developed to enable living cellular and microvascular structures visualized with magnification level up to 1,000-fold and to provide better spatial resolution than conventional fluorescence microscopy [31]. A low-powered blue laser light is emitted and focused onto a point of interest in a defined microscopic field. The emanating light from the observed point is focused to a pin hole which rejects out-of-focus light and avoids contamination by light scattering from different focal planes. After passing the pin hole, the fluorescent light projects to a photodetection device and in turn transforms into electronic signals. Because the illumination and detection systems are in the same focal plane, the endomicroscopy is termed “confocal.” All detected transformed electronic signals from the illuminated spot are measured and computed. Exogenous fluorescence contrast agents, such as fluorescein, acriflavine, or cresyl violet, are needed either in systemic or topical application to generate CLE images. The most common contrast agents are topical spraying acriflavine hydrochloride (0.05% in saline) or intravenous fluorescein sodium (5–10 mL of a 10% solution). Two kinds of CLE devices are commercialized available: integrated into an endoscope (eCLE) (Pentax, Tokyo, Japan) and as a stand-alone probe (pCLE) capable of passage through the accessory channel of most endoscopes (Cellvizio, Mauna Kea Technologies, Paris, France) [32]. 

 Endocytoscopy (EC) (Olympus, Tokyo, Japan) was developed using the principle of contact light microscopy with ultra-high magnification at 1,000- to 1,400-fold level [33–35]. In contrast to CLE which can visualize mucosal structures up to 250 *μ*m below the surface layer, EC only allows visualization of the very superficial (50 *μ*m) mucosal layer. After treating the mucosa with mucolytic agents, such as N-acetylcysteine, mucosal staining with 1% methylene blue in the oesophagus and with 0.25% toluidine blue in the stomach and colon is sprayed for EC examination. About 60 seconds of exposure to the dye, repeat instillation of the mucosa is needed to remove excess dye before ultra-high magnification examination. The instruments of EC include probe-based (pEC) and endoscope-based (iEC) systems. The pEC system has 2 flexible catheter devices that provide ultra-high magnification imaging of the epithelial surface at 570-fold or 1400-fold on a 19-inch monitor (or 450-fold and 1125-fold on a 14-inch monitor). The iEC system uses two separated lenses and is integrated into an 80-fold magnification endoscope enabling 450- to 580-fold magnification on a 19-inch monitor [33, 35].

The observation at the level of virtual histology by the CLE and EC techniques offers real-time *in vivo *“optical or virtual” biopsy. These technological advances of endoscopy equipment might replace traditional endoscopy-guided biopsy which is sometimes insufficient to make correct diagnosis.

#### 2.1.5. Optical Coherence Tomography

 Optical coherence tomography (OCT) is a cross-sectional mapping of optical reflectivity by means of infrared light based on the principles similar to B-mode ultrasound [36]. The high resolution around 15~20 *μ*m makes acquisition of imaging nearly at the histology level possible. An optical probe with fiber optic and electrical cable in a flexible tube, and lateral scanner with lens at the distal end, is inserted into the working channel of endoscope with contact of target tissue. The targeted tissue is discriminated as layers determined by the time it takes for the infrared light to contact various layers of the intestinal wall and reflect back to the detector, optical interferometry [37]. The light is split into sampling and reference arms; the latter reflects from a mirror and returns to the same point where it originated then recombines with the sampling light. These two arms interfere, producing oscillations or fringes. By measuring the amplitude of oscillations and the coherence length, a function of depth and lateral coordinates can be computed to produce OCT images.

#### 2.1.6. i-Scan

 The i-scan technology utilizes the digital contrast method to produce high resolution enhanced images. Three kinds of image enhancement are available, that is, surface enhancement (SE), contrast enhancement (CE), and tone enhancement (TE) [38, 39]. The SE mode enhances the light contrast by obtaining luminance intensity for each pixel, and edges of images are enhanced for more extensive observation of the glandular structures of mucosa surface. The CE mode adds blue light component and slightly suppresses the red and green components in lower luminance intensity area. As a result, the bluish-white discoloration of the lower luminance areas is produced for detailed observation of subtle irregularities of mucosal surface. In the TE mode, the RGB components of conventional endoscopic images are separated into each light component, and each isolated component is converted independently along the S or J tone curve by modification of input and output parameters, followed by reconstruction of each light component to produce TE images. The J type tone curve makes structural changes clearer by suppressing the maximal output of R component and enhancing overall GB component. The S type tone curve shifts the high R component area to a higher range or the low R component region to a further lower range of R to enhance the sensitivity to GB components. So far, about 6 types of TE are available for different tone enhancement for specific condition: TE-p (enhanced R component), TE-v (suppressed R component similar to NBI system), TE-b (for BE), TE-e (for esophagus), TE-g (for stomach), and TE-c (for intestines) [38]. The i-scan system can provide detailed observation of mucosal surface by digital contrast method with simple and quick pushing-button switchover.

#### 2.1.7. Chromoendoscopy

**Figure 3:**
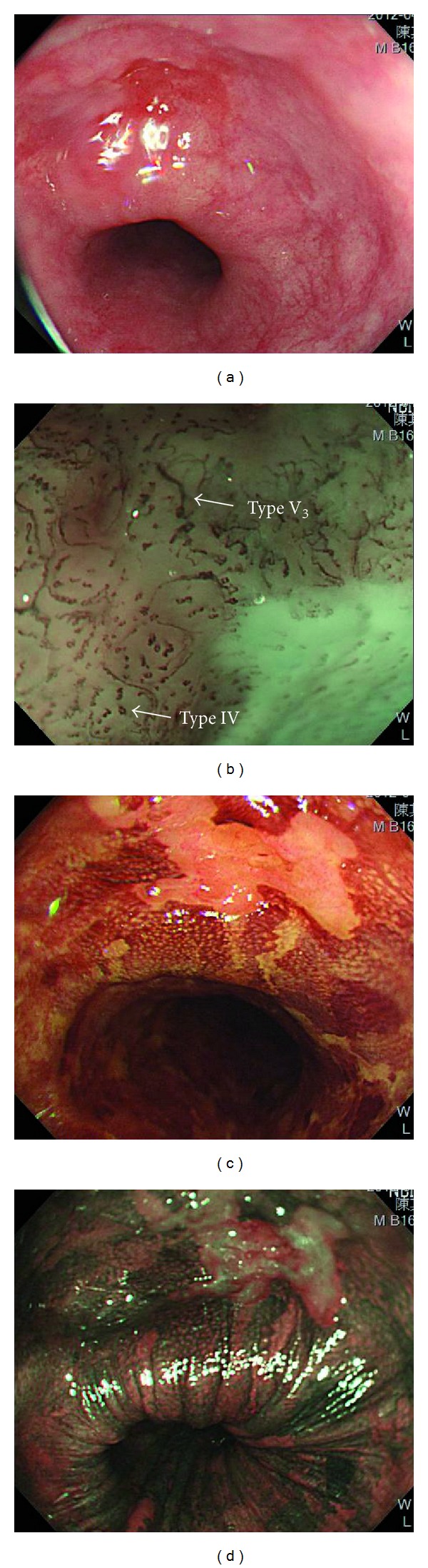
(a) Conventional white-light imaging endoscopy shows a type 0-IIc hyperemic lesion of esophagus. (b) Magnifying endoscopy with narrow-band imaging system reveals abnormal intraepithelial papillary capillary loops (Inoue's classification type IV to V_3_). (c) and (d) Chromoendoscopy with 1.5% Lugol's solution discloses Lugol-unstained area which appears pinkish under white-light imaging and silver pattern under narrow-band imaging 3 minutes after spraying dyes.

**Figure 4:**
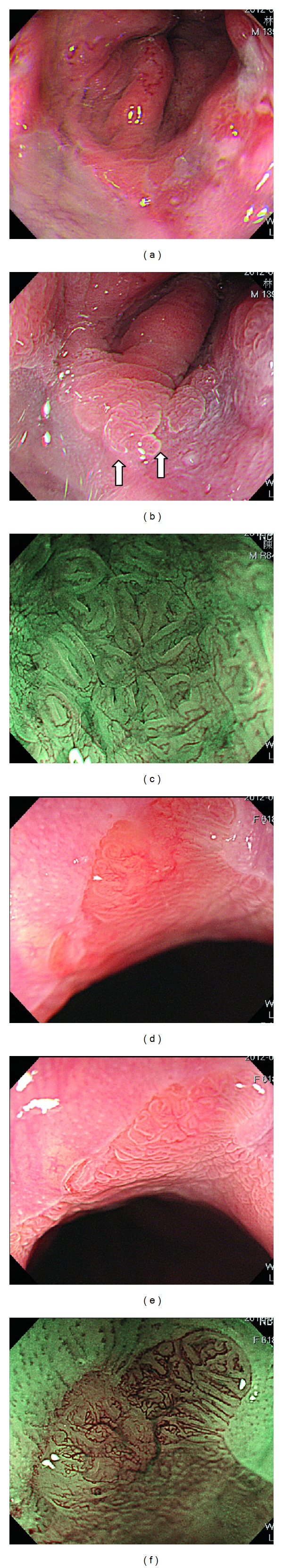
(a) White-light imaging endoscopy shows Barrett's esophagus. (b) Chromoendoscopy with 2% acetic acid shows “acetowhitening” reaction of the mucosa with intestinal metaplasia. (c) Magnifying endoscopy with narrow-band imaging system reveals nondysplastic mucosa presenting cerebriform or gyri-like pit pattern with superficial blood vessels regularly situated between the mucosal ridges. (d) Magnifying endoscopy with white-light imaging shows increased vascularity of mucosa breaks which appears as a villous pit pattern (e) after acetic acid spraying. (f) Magnifying endoscopy under narrow-band imaging system disclosed low-grade dysplasia with irregular/disrupted mucosal patterns and irregular vascular patterns.

 The most commonly used dyes for UGI tract mucosa are Lugol's solution, indigo carmine, acetic acid, and methylene blue [40]. Dyeing chromoendoscopy has been introduced over 40 years ago to enhance contrast differences between normal and neoplastic mucosa. For squamous epithelium of the esophagus, 0.5~5% Lugol's solution which is composed of iodine and potassium iodide can be well absorbed by glycogen-containing normal squamous epithelium showing brownish discolored “silk crape” like surface [40, 41]. The dysplastic/cancerous squamous epithelium with less glycogen do not stain and appears white-yellowish Lugol-voiding areas. If a light-pink part (silver discoloration under NBI system) appears in the iodine-unstained region within 3 minutes after spraying, the lesion is highly suspected as HGIN (Figure 3) [42]. The pink-silver sign has the sensitivity and specificity of 91.9% and 94.0%, respectively, to diagnose HGIN or invasive carcinoma [42]. However, certain side effects may develop after spraying Lugol's solution to esophagus, including chest pain, retrosternal cold sensation, nausea, or generalized itching allergic reactions [43]. To avoid unpleasant side effects, usually the concentration of 1.5–2% is sufficient to obtain adequate contrasting images. Sodium thiosulfate spraying after Lugol's chromoendoscopy can relieve irritation by Lugol's solution [43]. The 0.2~0.4% indigo carmine solution can accentuate the border and surface topography by pooling into the crevices of the mucosal surface. The margin and mucosal pattern of the columnar epithelium of stomach and intestines can be well delineated by chromoendoscopy with indigo carmine solution. Using 1~3% acetic acid for chromoendoscopy, “aceto-whitening” reaction may develop due to reversible acetylation of nuclear proteins (Figure 4). This reaction only lasts a few minutes and is even more quickly lost in dysplastic/cancerous regions which become red faster than nondysplastic BE [44]. The methylene blue dye composed of methylthioninium chloride can be absorbed by absorptive epithelial cells of small intestine, colon, and intestinal metaplasia at any site of the gastrointestinal tract which are stained blue. By spraying 0.1~0.5% methylene blue, specialized intestinal metaplasia (SIM) is stained as the presence of dark blue mucosa that persists despite vigorous irrigation, whereas staining pattern heterogeneity and decreased stain intensity suggest Barrett's high-grade dysplasia or cancerous changes. However, methylene blue chromoendoscopy should be cautiously performed because carcinogenesis risk could be increased due to oxidative damage to DNA by the photosensitized dye after white-light exposure [45]. The clinical data supporting the potential carcinogenic effect of methylene blue is still inconclusive and needs further investigation [46].

## 3. The Application of IEE in Esophagus

### 3.1. Narrow-Band Imaging System with Magnifying Endoscopy

#### 3.1.1. Squamous Cell Carcinoma of the Esophagus

Esophageal neoplasia can be detected as brownish discoloration areas by the NBI system because of its characteristic of hypervascularity [22, 24]. By endoscopic screening with NBI system in high-risk population, the prevalence of advanced esophageal neoplasia could be as high as 28% [47, 48]. Even in head and neck cancer patients with trismus and difficulty in oral intubation of standard endoscopy, the use of ultrathin transnasal endoscope equipped with NBI system is feasible for esophagus screening (sensitivity 88.9% and specificity 97.2% for high-grade intraepithelial neoplasia (HGIN) and invasive carcinoma) [49]. By ME-NBI system observation of esophagus, the capillaries derived from the branching vessels in the submucosa extending to the epithelial layer appear as dark brownish tennis racket-shaped dots. These microvascular structures are named as “intraepithelial papillary capillary loops” (IPCLs) [25]. Because of angiogenesis in ESCC, the changes in morphology with four characteristics, including dilatation, tortuosity, meandering enlarged caliber changes, and variation in shape of IPCLs, are observed [24, 50, 51]. Brownish discoloration of background epithelium under ME-NBI was also significantly (OR 25.5, 95% CI: 2.4–268) associated with mucosal high-grade dysplasia [52]. According to the different IPCLs pattern, ME-NBI can clearly distinguish esophageal neoplasia from nonneoplasia [51].

**Figure 5:**
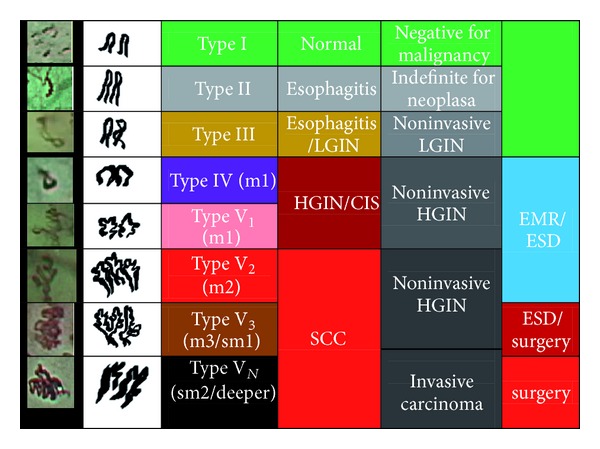
Inoue's classification of intraepithelial papillary capillary loops for esophageal neoplasia.

**Figure 6:**
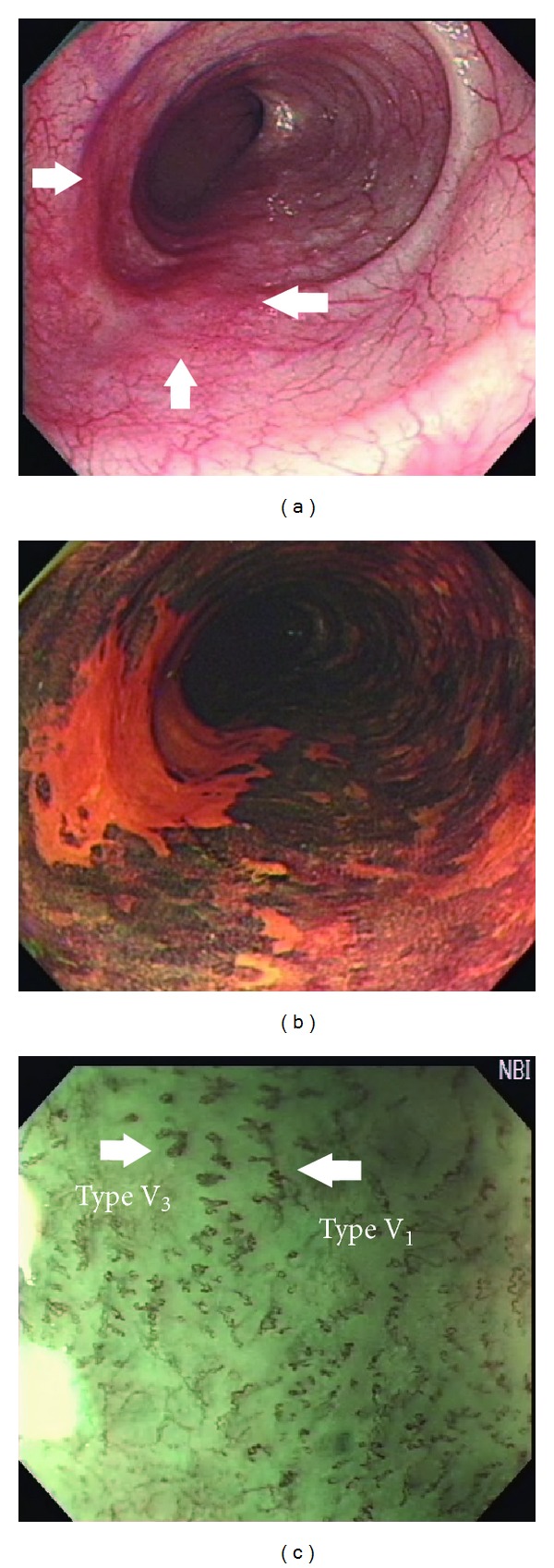
(a) White-light imaging endoscopy shows a type 0-IIb lesion with mildly hyperemic change of mucosal surface. (b) Chromoendoscopy after spraying 1.5% Lugol's solution discloses Lugol-unstained appearance. (c) Magnifying endoscopy with narrow-band imaging system reveals abnormal superficial vessels (Inoue's classification of intraepithelial papillary capillary loops type V).

**Figure 7:**
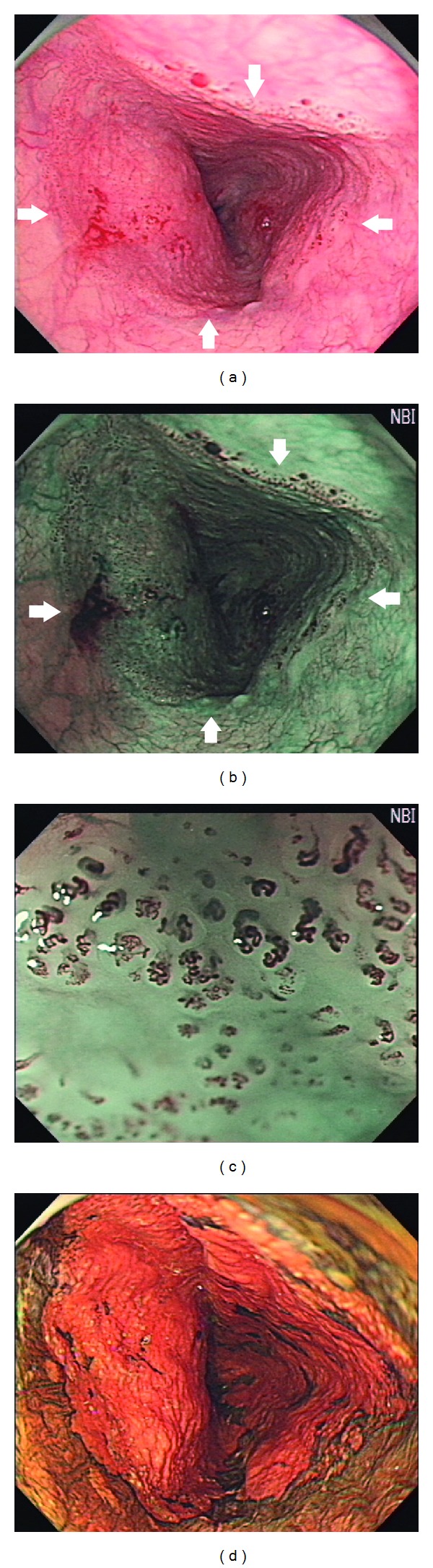
(a) Conventional endoscopy shows an esophageal circumferential long-segment neoplasia with hyperemic changes and nodularity of surface mucosa which turns to brownish discoloration (b) under narrow-band imaging system. (c) Under magnification with narrow-band imaging, abnormal superficial vessels are well demonstrated (Inoue's classification type V_*N*_). (d) Chromoendoscopy with 1.5% Lugol's solution shows extended Lugol-voiding area.

Inoue et al. further classified IPCLs into type I to V (Figure 5) [50, 53]. Type I IPCLs are observed in normal squamous epithelium. Type II IPCLs have elongation and/or dilatation changes and are often seen in esophagitis (Figure 1). Type III IPCLs which are iodine voiding under Lugol's solution staining have minimal color changes from type I IPCLs without proliferation, and these changes correspond to chronic esophagitis or low-grade intraepithelial neoplasia (LGIN). Type IV IPCLs have two to three of four morphology changes and are associated with HGIN or carcinoma *in situ*. Type V IPCLs demonstrate all four morphology changes. Type V IPCLs are further categorized from type V-1, V-2, V-3 to V_N_. In type V-1 IPCLs representing carcinoma *in situ* (m1), changes include dilatation with tortuosity, meandering irregular calibers, and variable forms (Figure 6). Type V-2 IPCLs which are found in cancer invading lamina propria (m2) are the extension form of V-1. Type V-3 IPCLs corresponding to cancer involving the muscularis mucosa (m3) or superficial submucosa (sm1) are advanced destruction form of capillaries running in a horizontal plane (Figure 6). As for the type V_N_ IPCLs (Figure 7), generation of large-caliber new tumor vessels which could be 10 times larger than the V-3 IPCLs appears in deeper submucosal cancer (sm2). The accuracy of depth prediction by Inoue's classification of IPCLs was about 83.3% [54].

Another classification system of IPCLs was proposed by Arima et al. [55]. IPCLs are categorized from type 1 to 4. Type 1 was characterized by thin, linear capillaries in the subepithelial papilla. Type 2 appears as regularly arranged vessels with dilatation and variable branching or spiral enlargement. Type 3 was characterized by destruction of crushed vessels with an irregular caliber. Type 4 was characterized by irregular multilayered, irregularly branched, or reticular vessels. According to Arima's classification system, most were normal mucosa (79.5) or inflammatory changes (15.4%) in type 1; 64.1% were inflammation and 14.1% were mild-to-moderate dysplasia in type 2; 86.9% were m1~m1 cancers in type 3; and 89.6% were m3 or deeper cancers in type 4. Moreover, the size of the avascular areas (AVAs) was closely associated with the cancer invading depth. Examination of the size of AVAs as well as the presence of stretched type 4 IPCLs can predict the extent and depth of HGIN or cancer well with correction rate up to 94.2% [55].

Identifing the depth of invasion and histology for superficial esophageal neoplasia is important for treatment strategy. Close periodically endoscopic surveillance is mandatory for LGIN of esophagus, and endoscopic resection should be done for HGIN of esophagus [8, 9, 16, 17, 24]. Given the low risk of LN metastasis for mucosal cancers of esophagus, ESD or EMR should be done for m1/m2 cancers with absolute indication and relatively applied to m3 cancer if *en bloc* resection is possible without evidence of lymphovascular invasion. To provide rapid real-time information of predicted histology and invasiveness of neoplasia, NBI system, especially in combination with ME, should be used in routine surveillance of esophageal cancer [8, 9, 24, 47, 51].

#### 3.1.2. Premalignant Lesions of Adenocarcinoma—Barrett's Esophagus

 High resolution magnifying endoscopy combined with NBI system can improve the detection of SIM and dysplasia of distal esophagus. By thorough examination of microstructure and microvascular patterns, the sensitivity, specificity, positive predictive value (PPV), and negative predictive value (NPV) of the combination of regular microstructural pattern (tubular/villous/linear) and absent microstructural pattern to detect SIM were 100%, 78.8%, 93.5%, and 100%, respectively [56]. The sensitivity, specificity, PPV, and NPV of the irregular microvascular/microstructural pattern for the prediction of HGIN were 90%, 100%, 99.2%, and 100%, respectively [56]. Singh et al. has developed a simplified grading system for BE by using ME-NBI examination [57]. The PPV and NPV for lesions with type A (round pits and regular microvasculature) for the histology of columnar mucosa without SIM were 100% and 97%, respectively; for type B (villous/ridge pits with regular microvasculature) or type C (absent pits with regular microvasculature) for histology of SIM, they were 88% and 91%, respectively and for type D (distorted pits with irregular microvasculature) for histology of HGIN 81% and 99%, respectively [57]. A recent meta-analysis has shown good diagnostic performance of ME-NBI system for BE [58]. For diagnosing HGIN, the pooled sensitivity, specificity, diagnostic OR, and area under the curve (AUC) were 96%, 94%, 342.49 (95% CI 40.49–2896.89), and 0.99, and for the characterization of SIM, the pooled sensitivity, specificity, diagnostic OR, and AUC were 95%, 65%, 37.53 (95% CI 6.50–217.62), and 0.88 [58]. By targeted biopsy of suspicious lesions identified by ME-NBI system, the number of random biopsies may be reduced and the diagnostic yield may be improved.

### 3.2. Flexible Spectral Imaging Color Enhancement

#### 3.2.1. Squamous Cell Carcinoma of the Esophagus

There are limited experiences in the application of FICE on the surveillance for ESCC. The method of selecting a suitable combination of wavelengths of RGB lights for esophageal squamous epithelium has not been well established. Inoue et al. have shown that the specific FICE modes A (R 550 nm, gain 2; G 500 nm, gain 2; B 470 nm, gain 3) and C (R 540 nm, gain 2; G 415 nm, gain 2; B 415 nm, gain 3) significantly enhanced the visibility of IPCLs of ESCC mucosa [27]. Some investigators have demonstrated a superior outcome in the diagnostic yield of esophageal capsule endoscopy by combination of the FICE system mode A with the PillCam ESO2 (Given Imaging, Yoqneam, Israel) [59]. However, although the microvessels could be visible more clearly by FICE system than conventional white-light imaging system, the relatively lower resolution of FICE than that of NBI system hampers further categorization of the microvascular morphology.

#### 3.2.2. Premalignant Lesions of Adenocarcinoma—Barrett's Esophagus

The identification of the palisading vessels of the esophagus and the termination of gastric folds is important to endoscopic diagnosis of BE. FICE system can improve visualization of the end of palisade vessels of the esophagus. Osawa et al. has demonstrated that the FICE system enables clear visualization of the demarcation of BE mucosa, gastric folds, and esophageal palisade vessels [60]. Pohl et al. has found that the FICE system has comparable diagnostic performance to acetic acid chromoendoscopy, with the sensitivity of targeted biopsy for HGIN/early cancer of 87% [61].

### 3.3. Autofluorescence Imaging System

#### 3.3.1. Squamous Cell Carcinoma of the Esophagus

AFI has higher accuracy for diagnosing early ESCC than WLI system, especially for flat/elevated lesions or those with diameter ≧20 mm, but ulcerations or inflammatory changes and depressed lesions or those with diameter <20 mm may cause misdiagnosis by AFI evaluation [29, 62]. Recently, a phase-II study in Japan has shown unsatisfactory diagnostic power of AFI system in screening esophageal HGINs, with sensitivity of 71% (95% CI 55–87%) and PPV of 25% (95% CI 16–34%), especially for lesions ≦10 mm [63]. Therefore, AFI system plays a limited role in screening ESCC, especially for those with small size and depressed morphology.

#### 3.3.2. Premalignant Lesions of Adenocarcinoma—Barrett's Esophagus

 Panjehpour et al. has conducted one of the earliest studies using laser-induced AFI system to detect HGIN in BE mucosa [64]. The analysis of the fluorescence spectra using the differential normalized fluorescence intensity at 480 nm index showed that 96% of nondysplastic BE samples were classified as benign, 100% LGIN samples as benign, 90% of HGIN samples as premalignant, and 28% of low-grade with focal high-grade dysplasia samples as premalignant. Differences in 5-aminolevulinic acid (10 mg/kg orally 3 hours before endoscopy) induced protoporphyrin IX fluorescence intensity at 635 nm can also be used to distinguish dysplastic to nondysplastic lesions with the sensitivity of 77% and specificity of 71% [65]. Although the AFI has high sensitivity and NPV, its strength is limited by the moderate specificity and PPV for detection of dysplasia in BE lesions [66]. Using NBI system as an adjunct to increase accuracy of detecting dysplasia, the false positive rate of AFI system can be reduced from 40~81% to 10~48% [66–68]. Thereby, endoscopic trimodal imaging (ETMI) system incorporating high-resolution endoscopy, AFI, and NBI systems has been developed to increase detection of HGIN of BE [68]. However, recent randomized multicenter crossover studies have shown that the yield of targeted biopsies of ETMI was significantly inferior to the overall yield of standard video endoscopy with 4-quadrant random biopsies every 2 cm [67, 69]. Moreover, the interobserver agreement for AFI suspected lesions is only fair to substantial (*κ* = 0.62 for experts and *κ* = 0.28 for nonexperts). At present, ETMI seemingly cannot replace random biopsies by standard video endoscopy for detection of dysplastic lesions.

### 3.4. Confocal Laser Endomicroscopy and Endocytoscopy

#### 3.4.1. Squamous Cell Carcinoma of the Esophagus

 CLE can be used in differentiating neoplastic lesions from normal epithelium. IPCLs could be demonstrated by CLE (Pentax EC-3870 CIFK, Pentax, Tokyo, Japan). The superficial ESCC has higher proportion of irregular arrangement of epithelial cells. (79.4% versus 10.0%, *P* < 0.001), increased diameter of IPCLs (26.0 *μ*m versus 19.2 *μ*m, *P* < 0.001), and irregular shape IPCLs (82.4% versus 36.7%, *P* = 0.0002) than normal mucosa by CLE examination [70]. By the defined criteria for cellular (dark cells with different sizes and irregular architecture, without clearly visible borders) and vascular (twisted and irregular vessels, elongated capillaries with leakage of fluorescein) changes, CLE has an overall accuracy of 95% and the sensitivity and specificity of 100% and 87%, respectively, for diagnosing early ESCC [71]. However, the proportion of images with good quality was still unsatisfactory (less than 40%), and the interobserver agreement was substantial [70, 71]. 

EC observation could potentially replace the role of histologic examination on biopsied specimens in the diagnosis of ESCC. Inoue et al. used iEC type XGIF-Q260EC1 (Olympus Medical Systems Corp. Tokyo, Japan) which provides 450-fold magnification power and observation area about 400 × 400 *μ*m^2^ to *in vivo* evaluate tissue atypia of the esophagus [72]. Endocytoscopic atypical (ECA) was classified into five grades: ECA 1- large, cytoplasm—rich regularly arranged cells with a rhomboid shape (normal); ECA 2- round cells with different-sized small nuclei (inflammatory changes); ECA 3- small size but the nuclei are still compact (borderline lesions); ECA 4- higher cell density with an increased N/C ratio (suggestive of malignant lesions); ECA 5- irregularly arranged cells with various sizes with a high N/V ratio (definitely malignant lesions) [72]. By this grading system, the overall accuracy to differentiate between nonmalignant and malignant issues was 82%. Kumagai et al. further categorized the EC findings into Type 0 to 3: Type 0- normally stained with iodine solution; Type 1- unstained with iodine, but showing normal squamous epithelial cells (a low cell density with a low N/C ratio without nuclear abnormality); Type 2- unstained with iodine, showing a high cell density but no evident nuclear abnormality; Type 3- unstained with iodine, but with evidently increased nuclear density and abnormality [73]. There were high degrees of agreement for Type 1 (90.9% with normal or inflammatory changes) and Type 3 (92.3% with HGIN or invasive carcinoma) lesions with the histologic diagnosis, especially using the pEC type XEC120U (Olympus Medical Systems Corp. Tokyo, Japan) which provides 1125-fold magnification and a tissue field of view measuring 120 × 120 *μ*m^2^ [73]. 

CLE and EC are promising *in vivo* optical biopsy tools; however, overcoming technical problems concerning sufficient image quality and universal criteria for optical histologic diagnosis is essential before popular clinical application [31, 74]. 

#### 3.4.2. Premalignant Lesions of Adenocarcinoma—Barrett's Esophagus

CLE can be used to distinguish between different types of epithelial cells and delineate cellular and microvascular changes in BE epithelium. Earlier study conducted by Kiesslich et al. has shown that BE and associated neoplasia can be predicted by CLE with a sensitivity of 98.1% and 92.9% and a specificity of 94.1% and 98.4%, respectively (accuracy, 96.8% and 97.4%) [75]. A prospective randomized, double-blinded, controlled crossover trial disclosed that CLE with targeted biopsy doubled the diagnostic yield for neoplasia and was equivalent to the standard 4-quadrant biopsy procedure for the diagnosis of neoplasia, and nearly two-thirds of patients did not need any mucosal biopsies to make final diagnosis [76]. Another large prospective international multicenter study has demonstrated that pCLE combined with high-definition (HD) WLI significantly improved the ability to detect neoplasia in BE patients compared to HD-WLI alone (sensitivity 34.2% versus 68.3%, *P* = 0.002) [77]. A consensus for standardization of pCLE image criteria, called “Miami classification”, has been introduced to describe diagnostic parameters to differentiate between normal squamous epithelium, nondysplastic BE, HGIN, and adenocarcinoma in BE [78]. However, some studies showed that poor positive predictive value (46~67%) for pCLE to evaluate neoplasia in BE and pCLE can only be possibly regarded as noninferior to standard endoscopic biopsy [79, 80]. Although emerging evidence supports the benefits of CLE application in BE, more data are needed to justify this novel approach in the clinical setting.

 Pohl et al. has assessed the accuracy of EC in correlation with histology to distinguish neoplasia from BE in premarked areas [81]. In this study, only 23% of images with lower magnification (450-fold) were interpretable to identify characteristics of neoplasia and 41% with higher magnification (1125-fold). PPV and NPV for HGIN or cancer were 0.29 and 0.87, respectively, for 450-fold magnification and 0.44 and 0.83, respectively, for 1125-fold magnification. Endoscopic histology using EC lacks sufficient image quality to currently assist in identifying neoplastic areas in BE, and there are needs for an initial macroscopic wide-field surveillance technique to identify suspicious areas.

### 3.5. Optical Coherence Tomography

#### 3.5.1. Squamous Cell Carcinoma of the Esophagus

 OCT is not used for screening but can provide information for T staging of ESCC. Hatta et al. have demonstrated using OCT for preoperative staging with high degree of overall accuracy (92.7%) (m1/m2, 94.9%; m3, 85.0%; sm, 90.9%) [82]. Moreover, a nonrandomized comparative study has shown higher accuracy for m1/m2 cancer staging by using OCT than that by using EUS with 20-MHz miniature probe (94.6% versus 80.6%, *P* < 0.05) [83]. However, because of limited depth of penetration by OCT, the deeper submucosa and structures beyond the muscularis propria cannot be welldelineated. Further randomized prospective studies are needed to demonstrate the role of OCT in staging of superficial ESCC.

#### 3.5.2. Premalignant Lesions of Adenocarcinoma—Barrett's Esophagus

 OCT is a promising diagnostic tool for optical biopsy, and it can reduce the need for random biopsies with standard WLI endoscopy. Poneros et al. has defined OCT images for SIM with sensitivity and specificity of 97% and 92%, respectively, by the characteristics of (1) absence of the layered structure of normal squamous epithelium and the vertical “pit and crypt” morphology of gastric columnar epithelium, (2) distorted architecture with heterogeneous tissue contrast and an irregular surface, and (3) presence of submucosal glands [84]. A prospective double-blinded study has an accuracy of 78% for the detection of dysplasia in BE mucosa [85]. Using computer-aided diagnosis system for classification of BE mucosa by OCT imaging may yield higher accuracy of 84% for detection of dysplasia [86]. Moreover, the application of 3D-OCT imaging can provide real-time information for endoscopic ablation therapy response and identify residual dysplastic lesions which need further ablation [87]. OCT has the potential for diagnosing BE and differentiating dysplastic from nondysplastic lesions without histological biopsy.

### 3.6. i-Scan

**Figure 8:**
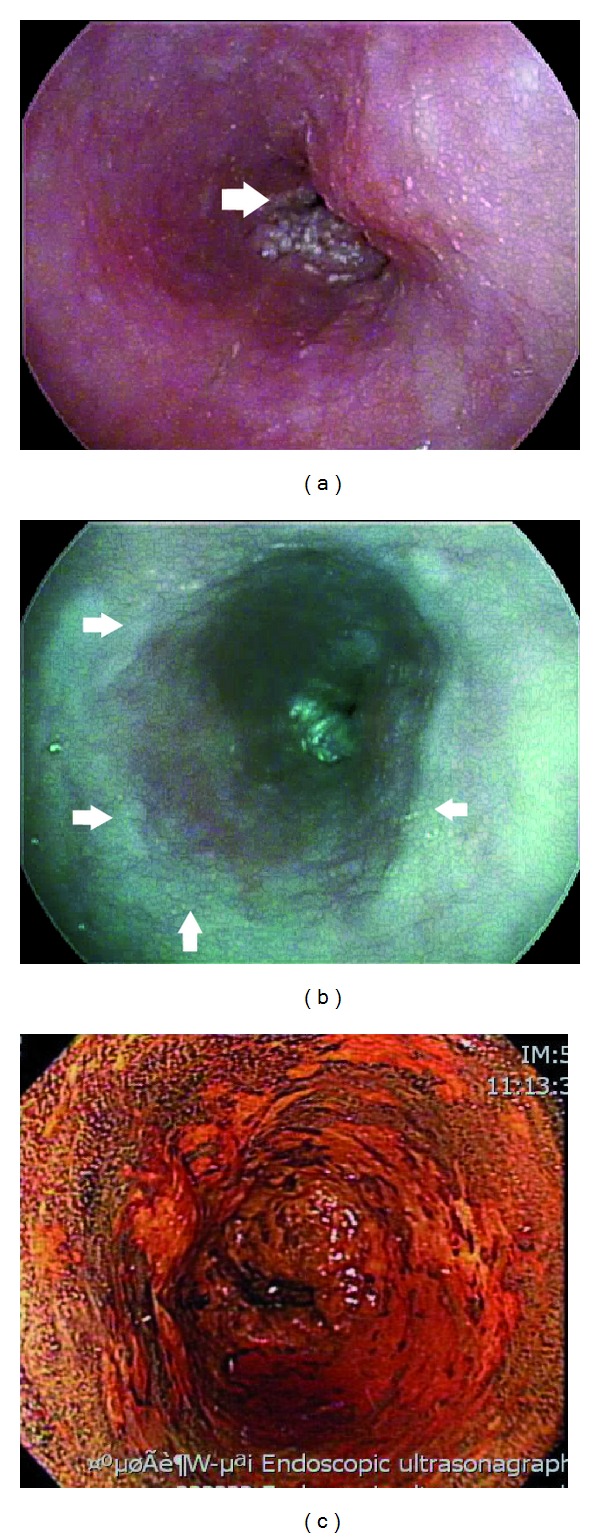
(a) Conventional white-light imaging endoscopy shows an ulcerative mass with lumen obstruction of the esophagus. (b) i-scan (SE 6+, CE 4+, TE-e) discloses reddish discoloration of the adjacent mucosa which is Lugol unstained (c), and the pathology is high-grade intraepithelial neoplasia.

There are limited data on the application of i-scan for screening of esophageal precancerous or cancerous lesions although premalignant mucosa of esophagus can be well delineated by i-Scan (Figure 8). However, i-scan can efficiently detect minimal changes of mucosa in patients with gastroesophageal reflux disease [88, 89]. 

### 3.7. Chromoendoscopy

#### 3.7.1. Squamous Cell Carcinoma of the Esophagus

By using chromoendoscopy with Lugol's solution for screening ESCC in high-risk population, such as alcoholics or head and neck cancer patients, the prevalence of HGIN or invasive carcinoma of the esophagus could range from 3.2% to 16.6% [47]. As long as the concentrations of cellular glycogen content change, differences in degree of iodine staining enhance the contrast of the abnormal squamous epithelium. However, because lesions with chronic inflammation, squamous hyperplasia, or LGIN could be iodine unstained, Dawsey et al. has found that the chromoendoscopy with Lugol's solution has high sensitivity (96%) but low specificity (63%) for identifying HGIN or invasive carcinoma of esophagus [90]. Lee et al. has also demonstrated lower diagnostic performance of chromoendoscopy with Lugol's solution (sensitivity 88.9%, specificity 72.2%) than that of NBI system (sensitivity 88.9%, specificity 97.2%) in ESCC screening [49]. Nevertheless, to well delineate the spread of superficial esophageal neoplasia, especially for flat or slightly depressed lesions, iodine staining by spraying Lugol's solution before EMR or ESD is essential to mucosa marking of endoscopic resection margin.

#### 3.7.2. Premalignant Lesions of Adenocarcinoma—Barrett's Esophagus

 The dyes mainly applied to the survey of BE are methylene blue, acetic acid, and indigo carmine. The sensitivity, specificity, and overall accuracy of methylene blue for detecting SIM is 98%, 61%, and 95%, respectively [91, 92]. Endo et al. categorized BE pit patterns by ME-WLI into five types: small round, straight, long oval, tubular, and villous pit patterns [93]. The tubular and villous pit patterns which are positive staining for methylene blue are closely associated with the presence of SIM. Although a previous randomized crossover study has shown superior diagnostic accuracy (75% versus 68%, *P* = 0.032) of the methylene blue directed biopsy technique to that of the random biopsy technique for identifying SIM [94], a recent meta-analysis has conflicting result [95]. The technique of methylene blue chromoendoscopy has only comparable yield with random biopsy for the detection of SIM and dysplasia [95].

 A significant improvement in detecting SIM of esophagus by acetic acid chromoendoscopy has been documented. Based on the acetowhitening reaction of columnar epithelium, chromoendoscopy with acetic acid can enhance the architecture of BE epithelium. The whitening reaction is lost in dysplastic tissues earlier than nondysplastic mucosa, aiding further distinguishing neoplasia from normal epithelium. A prospective randomized crossover study has shown that acetic acid-guided biopsies with the adjunct of ME were superior to standard video endoscopy with random biopsies (78% versus 57%), and the number needed to confirm BE was reduced [96]. ME with acetic-acid staining had an accuracy of 83.8% for prediction of BE [96]. Even without magnifying, acetic-acid chromoendoscopy can identify dysplasia or cancer in BE with the sensitivity and specificity of 95.5–100% and 80–97.7%, respectively [97, 98]. 

## 4. The Application of IEE in Stomach

### 4.1. Narrow-Band Imaging System with Magnifying Endoscopy

#### 4.1.1. Helicobacter pylori-Associated Gastritis

NBI alone [99], HD-ME [100], and ME-NBI [101] all had good correlation between histopathological findings and Hp-associated gastritis, atrophic gastritis, and IM. HD-ME can reliably identify the normal gastric mucosa, Hp-associated gastritis, and gastric atrophy [100]. By identifying changes in morphology of subepithelial capillary network (SECN), connecting venules and mucosal pits, the sensitivity, specificity, PPV, and NPV for predicting an Hp-infected stomach and gastric atrophy were 100% (95% CI 83.9–100%), 90% (95% CI 66.8–98.2%), 92.7% (95% CI 93.2–97.3%), 96% (95% CI 87.9–98.9%), and 83.8% (95% CI 65.5–93.9%), 85.7% (95% CI 62.6–96.2%), 100% (95% CI 92.9–100%), and 97.3% (95% CI 89.6–99.5%), respectively [100]. The magnified views of Hp-related gastritis significantly differed from normal mucosa presenting collecting venules and true capillaries forming a network with gastric pits in the center [102]. With the adjunct of NBI system to HD-ME, the mucosal pit pattern and microvascular architecture could be visualized more clearly. Hp-infected stomach and the histological severity of gastritis and atrophy can be predicted accurately [101].

#### 4.1.2. Gastric Intestinal Metaplasia

**Figure 9:**
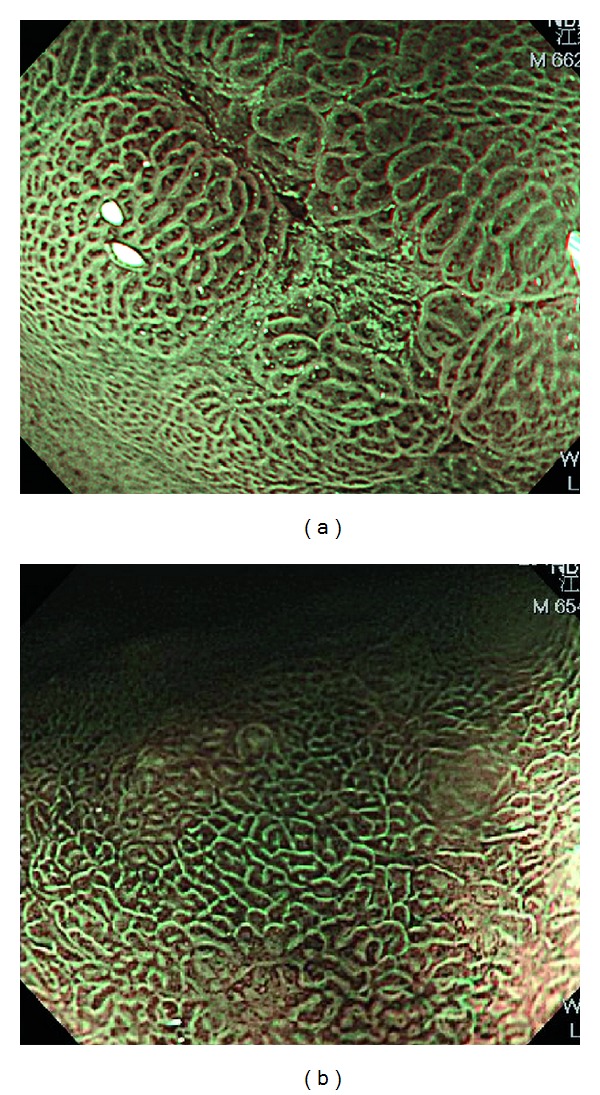
Magnifying endoscopy with narrow-band imaging of intestinal metaplasia change of gastric mucosa shows light blue crest sign (a fine, blue-white line on the crests of the epithelial surface).

Gastric IM is a risk factor of intestinal-type gastric cancer, but WLI was not adequate to detect IM of stomach [103]. NBI system with and without magnification can excellently diagnose GIM with good histological agreement. Uedo et al. used a light blue crest (LBC) on epithelial surface by ME-NBI as a maker to diagnose IM with the sensitivity and specificity of 89% and 93%, respectively [104]. The LBC is a fine, blue-white line on the crests of the epithelial surface (Figure 9), and it can only be detected under wavelengths of 400–430 nm. A multicenter study showed that regular ridge or tubulovillous mucosa can accurately predict IM with the sensitivity, specificity, and accuracy of 89%, 90%, and 90%, respectively [105].

#### 4.1.3. Early Gastric Cancer

Conventional endoscopy has the limitation in detecting lesions by morphological changes, and the diagnosis of malignancy depends on pathology. Few morphologic changes could be detected by WLI to differentiate malignant from nonmalignant lesions in EGC. The minute surface structure and microvessels observed by ME were related to histopathological findings [106]. Combining NBI system with ME could maximize the benefit for diagnostic accuracy, such as microvascular architecture, microsurface structure, and demarcation line (DL) between cancer and surrounding mucosa [107, 108]. Kaise et al. used the triad ME-NBI findings, including fine mucosal surface disappearance, microvascular dilation, and heterogeneity, to diagnose EGC with sensitivity and specificity of 69.1% and 85.3%, respectively, and the AUC of ROC in multivariate analysis was 0.86 [108]. Yao et al. described a simple classification system, called “VS classification”, by regularity and presence of vascular and microsurface patterns under ME-NBI [109]. In this study, irregular vascularity and irregularity or absence of microsurface pattern with well-delineated DL were highly associated with carcinomatous gastric mucosa [109]. Sometimes, white opaque substance (WOS) within epithelium obscures observation of microvascular pattern under ME-NBI. WOS can be seen more frequently in adenoma than in carcinoma (78% versus 43%) [110]. For gastric neoplasia of type 0-IIa type with either WOS with a regular distribution or a regular microvascular pattern, the sensitivity and specificity for discriminating adenoma from carcinoma were 94% and 96%, respectively [110].

### 4.2. Flexible Spectral Imaging Color Enhancement

Mouri et al. has analyzed the wavelengths to generate the maximum difference of the spectral reflectance between the normal gastric mucosa and the EGC, and the result has shown that setting the wavelength at 530 nm of FICE observation resulted in an improvement in the visualization of the EGCs [111]. The diagnostic accuracy of extent of gastric cancer using FICE is superior to that using conventional WLI system [112], and with selection of specific reflectance spectrum, FICE is useful in discriminating among nonneoplastic lesions, adenoma, and EGC [28].

### 4.3. Autofluorescence Imaging System

An earlier study showed that AFI had high sensitivity (96.4%) but low specificity (49.1%) to diagnose gastric neoplasm [113]. Uedo and his coworkers used a case series study to conclude that AFI had lower diagnostic accuracy (68%) than that of chromoendoscopy (91%), but with an advantage over standard WLI (36%) in diagnosis of EGC [29]. Moreover, open-type, atrophic fundic gastritis diagnosed by AFI was significantly associated with the development of metachronous gastric cancer (hazard ratio 4.88, 95% CI 1.32–18.2) after Hp eradication therapy [114]. However, there are some limitations for AFI to diagnose EGC accurately. Ulcerations, inflammation, or scars may cause overdiagnosis in the AF observation [29], and AFI tends to overestimate the size of gastric neoplasm [115].

### 4.4. Confocal Laser Endomicroscopy and Endocytoscopy

#### 4.4.1. Hp-Associated Gastritis

CLE with topical acriflavine was firstly used to identify *Hp* infection in a patient in 2005, and the bacterium which uptakes acriflavine *ex vivo* was seen as bright dots [116]. A prospective study uses three features under acriflavine-guided CLE, including white spots, neutrophils, and microabscesses, to detect *Hp* infection with the accuracy, sensitivity and specificity, of 92.8%, 89.2%, and 95.7%, respectively [117]. According to the established CLE criteria added with the presence of fluorescein leakage, histological severity of Hp-associated gastritis was graded [118]. The sensitivity and specificity of CLE were 82.9% and 90.9% for the diagnosis of Hp infection, 94.6% and 97.4% for predicting gastric normal mucosa, 98.5% and 94.6% for predicting active inflammation, 92.9% and 95.2% for predicting atrophy of glands, and 98.6% and 100% for diagnosing IM, respectively [118].

#### 4.4.2. Gastric Intestinal Metaplasia

 A prospective study used the histopathological criteria to diagnose GIM by CLE. These criteria included goblet cells, columnar absorptive cells and brush border, and villiform foveolar epithelium [119]. CLE can differentiate complete metaplasia (sensitivity/specificity 68.03/89.66%) from incomplete metaplasia (sensitivity/specificity 68.42/83.41%), with the latter being closely associated with gastric cancer, according to the shape of the goblet cells, the presence of absorptive cells or brush border, and the architecture of vessels and crypts [119]. In another prospective study conducted by the same group, CLE found out 189 patients with gastric IM in 1572 “endoscopic normal-looking mucosa” patients by WLI system [120]. Real-time iCLE diagnosis had a higher sensitivity (88.9%), specificity (99.3%), and accuracy (98.8%) for gastric superficial cancer/HGIN lesions than WLI diagnosis (sensitivity, 72.2%; specificity, 95.1%; accuracy, 94.1%) (*P* < 0.05) [120].

#### 4.4.3. Early Gastric Cancer

 Kakeji et al. reported *ex vivo* tissues of 27 gastric cancers using CLE had sensitivity, specificity, and accuracy of 88–92.6%, 100%, and 94.4–96.3% (by endoscopists and pathologist), respectively [121]. Kitabatake et al. used *in vivo* CLE images to interpret by pathologists, and the results were compared with histology. The diagnostic accuracy was 94.2–96.2% [122]. A prospective comparative study has demonstrated higher accuracy of CLE diagnosis of gastric adenomas and adenocarcinomas than that of endoscopic biopsy (94.2% versus 85.7%, *P* = 0.031) [123]. The use of CLE optical biopsy could potentially replace conventional histological biopsy. 

 In field of EC for stomach, only a case report or a small sample size study was reported [124, 125]. Eberl et al. showed that the sensitivity and specificity of EC were lower in gastric lesions (56% and 89%) compared to those in esophagus and colon (about 79-80%, and 90–100%) [124]. The poor diagnostic performance of EC may come from gastric mucus secretion [124]. Good quality of high magnified images by CLE or EC cannot always be obtained and there is still a long way to go for clinical application in mass screening of GI tract neoplasia. 

### 4.5. Chromoendoscopy

Indigo carmine, a nonabsorbable dye, had been used in imaging of gastric lesions for more than 30 years, and it is still a useful technique to detect gastric neoplasia and determine the demarcation line before endoscopic resection [126, 127]. Acetic acid was also useful in distinguishing dysplastic from nondysplastic lesions based on the difference in duration of acetowhitening reaction between neoplastic and nonneoplastic mucosa. The whitening reaction disappeared less than 5 seconds in invasive carcinoma compared to that about 90 seconds in nonneoplastic mucosa and low-grade adenoma [128]. Tanaka et al. further categorized gastric lesions into five types according to enhanced-magnification endoscopy findings following 1.5% acetic acid instillation, and all of the signet-ring cell carcinomas and poorly differentiated tubular adenocarcinomas have shown irregular arrangement or destructive surface pattern [129]. When combining chromoendoscopy with zoom endoscopy, the microsurface and microvascular structures could be visualized more easily. Magnification chromoendoscopy with 1% methylene blue was validated to assess premalignant gastric lesions (chronic atrophic gastritis with or without intestinal metaplasia) with sensitivity and specificity of 100% and 99%, respectively [130]. Dinis-Ribeiro et al. also used magnification chromoendoscopy with methylene blue to classify pit pattern of gastric lesions into 10 subgroups [131]. The classification system had the specificity and NPV of 81% and 99%, respectively, to diagnose dysplasia. Congo red alone, or combined with methylene blue, has also been applied in chromoendoscopy [132, 133]. The correct diagnosis of synchronous multiple loci of EGC by Congo red-methylene blue chromoendoscopy could be higher than that of conventional WLI endoscopy (28.3% versus 88.9%) [133]. Prolonged procedure time by staining method is still the common problem for chromoendoscopy screening of GI neoplasia.

## 5. Conclusions

The early detection of precancerous or early cancerous lesions of gastrointestinal tract is of utmost importance to timely curative treatment, improved overall outcome, and the maintenance of a good quality of life. Therefore, visualizeing the invisibles by endoscopists based on the utilization of IEEs on cancer screening could solve problems from missing lesions by conventional WLI endoscopy. Optical imaging technology has high diagnostic accuracy, and image reconstruction technique could provide similar imaging with histopathological findings. After obtaining universal standardized imaging method at high magnification level and images with good quality, optical biopsy could replace histological biopsy in the near future.
